# Test-retest reliability of Brazilian version of Memorial Symptom Assessment Scale for assessing symptoms in cancer patients

**DOI:** 10.1590/S1679-45082017AO3645

**Published:** 2017

**Authors:** Josiane Roberta de Menezes, Bianca Maria Oliveira Luvisaro, Claudia Fernandes Rodrigues, Camila Drumond Muzi, Raphael Mendonça Guimarães

**Affiliations:** 1 Instituto Nacional de Câncer José de Alencar Gomes da Silva, Rio de Janeiro, RJ, Brazil.; 2 Fundação Oswaldo Cruz, Rio de Janeiro, RJ, Brazil.

**Keywords:** Neoplasms, Symptom assessment, Validation studies, Reproducibility of results, Scales

## Abstract

**Objective:**

To assess the test-retest reliability of the Memorial Symptom Assessment Scale translated and culturally adapted into Brazilian Portuguese.

**Methods:**

The scale was applied in an interview format for 190 patients with various cancers type hospitalized in clinical and surgical sectors of the *Instituto Nacional de Câncer José de Alencar Gomes da Silva* and reapplied in 58 patients. Data from the test-retest were double typed into a Microsoft Excel spreadsheet and analyzed by the weighted Kappa.

**Results:**

The reliability of the scale was satisfactory in test-retest. The weighted Kappa values obtained for each scale item had to be adequate, the largest item was 0.96 and the lowest was 0.69. The Kappa subscale was also evaluated and values were 0.84 for high frequency physic symptoms, 0.81 for low frequency physical symptoms, 0.81 for psychological symptoms, and 0.78 for Global Distress Index.

**Conclusion:**

High level of reliability estimated suggests that the process of measurement of Memorial Symptom Assessment Scale aspects was adequate.

## RESUMO

**Objetivo:**

Avaliar a confiabilidade teste-reteste da versão traduzida e adaptada culturalmente para o português do Brasil do *Memorial Symptom Assessment Scale*.

**Métodos:**

A escala foi aplicada em forma de entrevista em 190 pacientes com diversos tipos de câncer internados nos setores clínicos e cirúrgicos do Instituto Nacional de Câncer José de Alencar Gomes da Silva e reaplicada em 58 pacientes. Os dados dos testes-retestes foram inseridos num banco de dados por dupla digitação independente em Excel e analisados pelo Kappa ponderado.

**Resultados:**

A confiabilidade da escala mostrou-se satisfatória nos testes-retestes. Os valores do Kappa ponderado obtidos para cada item da escala apresentaram-se adequados, sendo o maior item de 0,96 e o menor de 0,69. Também se avaliou o Kappa das subescalas, sendo de 0,84 para sintomas físicos de alta frequência, de 0,81 para sintomas físicos de baixa frequência, de 0,81 também para sintomas psicológicos, e de 0,78 para Índice Geral de Sofrimento.

**Conclusão:**

Altos níveis de confiabilidade estimados permitem concluir que o processo de aferição dos itens do *Memorial Symptom Assessment Scale* foi adequado.

## INTRODUCTION

Symptoms are multidimensional experiences that include perception of frequency, intensity, distress and meaning of its occurrence and expression. A symptom can influence in the occurrence and meaning of other symptoms.^1^

When three or more symptoms occur concomitantly (*e.g.*, pain, fatigue, sleeping disorders, nausea, vomiting, and loss of appetite) they are associated and called a (cluster symptoms). These symptoms can be an side effect in development of the patient and they may cause a synergic effect, such as predictor of patient’s morbidity.^2^

Cancer is not a single disease, but a set of more than a hundred of different diseases.^3^ When the concept of group of diseases is established, the cancer becomes responsible to produce a variety of symptoms because of its complexity.

Malignant neoplasms is the second cause of death in the world and, according to perspectives, they should be considered the first cause of death in Brazil by 2020.^4^ Based on statistics of the Brazilian National Cancer Institute (INCA - *Instituto Nacional de Câncer José de Alencar Gomes da Silva*), approximately 596,000 cancer cases were expected in 2016 among Brazilian population.^5^

Malignant neoplasms are able to generate important physical and psychosocial changes, mainly because of appearance of symptoms that can be intensified along with development process of the disease. Inpatients with cancer have symptoms that vary in terms of severity, frequency and duration.^6^ Factors such as prevalence, intensity, and perception of symptoms impact life activities and they also present high variability among patients and are strongly influence by the disease itself and toxicity from the treatment.^7^ Symptoms evaluation constitutes, therefore, a challenge, because of evolution pathway and complex relation between the disease and symptoms.^8^

Each symptoms in oncology constitutes a dynamic phenomenon and, for this reason, symptoms should be always reevaluated in order to control intercurrences, and to provide relief and comfort to the patient.^9^ Considering the frequent occurrence of multiple symptoms in patients with cancer, there is the need of made available new validate instruments for assessment of prevalent symptoms, mainly for scarcity of tools in Brazil.

The Memorial Symptom Assessment Scale (MSAS) is an instrument develop in 1994 to provide multidimensional information about a diversified cluster of physical and psychological symptoms that are common in patients with cancer. This scale evaluates 32 physical and psychological symptoms, and its frequent dimensions, severity and distress by a Likert-type scale. In addition it provides an broadly assessment method for symptoms and can be useful when information about symptoms are desirable, such as in clinical trials or epidemiological studies.^10^

The validation of MSAS scale for Brazilian culture context is justified for the need of instruments to evaluate more broadly multiple symptoms in patients with cancer. This scale enables health professional to better understand the complexity of cluster of symptoms presented in a specific patients, or more frequently, in a specific type of cancer, therefore, this scale may guide health professionals and helps in development of interventions for management of such symptoms. This instrument can be also useful to be applied in epidemiological studies about cluster symptoms in oncology and quality of life.

The MSAS scale has been translated into Brazilian Portuguese and tested in terms of semantic equivalence. To establish psychometric properties of an instrument is important after its semantic adaptation.

Adapting process of an instrument is more than simple translation of words. It involves combination of translation from an idiom to another and standardized process that considers cultural context and life style of the target-population.^11^ Reichenheim et al.,^12^ suggest transcultural adaptation to be done in six phases: (1) concept equivalence; (2) equivalence of items; (3) semantic equivalence; (4) operational equivalence; (5) measurement equivalence; (6) functional equivalence.

Findings of this study indicated whether translated and adapted version was adequate to be used with oncology patients in Brazil.

## OBJECTIVE

To evaluate test-retest reliability of the Memorial Symptom Assessment Scale translated and culturally adapted into Brazilian Portuguese.

## METHODS

This study is the fifth stage of transcultural adaption of MSAS-BR scale that correspondent to the reliability assessment. As the first time we performed equivalence tests on concept, items, semantic, operational followed by the first four steps required in the process.^13^

### Characterization of the instrument

The MSAS scale was created in 1994 in the United States. It was constructed to detect and monitor symptoms of patients with cancer. This instrument was developed by oncology specialist and the scale combines physical and psychological symptoms with their degrees of severity, frequency and discomfort caused by symptoms.

The original version of the scale is composed by Likert-type scale including 32 symptoms. It is a self-reported instrument in which patients attribute a number from 1 to 4 for frequency and intensity of symptoms, from 0 to 4 for the degree of discomfort experienced during the last week at interview time. The responses order indicates a higher score meaning a worse clinical feature.

The stimulus is divided in subscales that evaluated psychological symptoms (PSYCH), with 6 items, physical symptoms of high frequency (PHYS H), with 12 items; and physical symptoms of relatively low frequency (PHYS L), with 14 items. Still there is a fourth subscale that contains four psychological symptoms and six physical symptoms in which global distress index (GDI) is evaluated, which may vary when applied, for example, ambulatory and hospitalized patients, and they can be considered more useful in a clinical point of view. Finally, there is final index that comprises the mean between three domains and all items (TMSAS). The internal consistence of these groups was evaluated in the original version by the Cronbach’s a coefficient, attributing the following values 0.835 to PSYCH, 0.882 to PHYS H and 0.580 to PHYS L.^10^

The MSAS version used in Brazil was translated and adapted by a group of specialists in Oncology and Epidemiology. The principal author of the original versions authorized by e-mail the translation and adaptation to Brazilian Portuguese. The semantic equivalence process of the MSAS-BR scale for Brazilian culture had satisfactory results and good acceptability by target-population during pre-tests. We observed high limitation for self-application of the instrument by target-population specially because educational *deficit* is still an issue in Brazil. For this reason, instrument was applied by interviewing. The 32 symptoms cited in the original instrument scale were maintained and their classification followed the same original score.^13^

### Data collection and analysis

Data were collected from March to December 2015. The MSAS-BR scale was applied to 190 patients at private interview after previous explanation about the objective of the study. We include those who agreed to participate and signed the consent form. The study population was composed by men and women patients, aged ran18 years or older, with multiple neoplasia and who hospitalized in clinical and surgical areas at *Hospital do Câncer* I (HCI/INCA). We excluded patients with cognitive disorders (neoplasia or metastasis of the center nervous system) that status could compromise the reliability of answers.

For additional data collection, we used our own sociodemographic and clinical data form including age, sex, marital status, level of education, race, primary diagnosis and metastasis.

A second interview was carried out and the scale was reapplied in 58 patients who remained hospitalized or were re-hospitalized in an interval between 5 to 15 days, from the first interview, and who had the same clinical condition and/or agreed to answer to re-test to check the scale reliability.

Answers of tests-retests were included in double typed spreadsheet from in Excel program, with posterior correction of inconsistences. Statistical data were analyzed. In assessment of individual variables, the stability analysis of test-retest of items and dimensions score was applied to Kappa statistics, in case of ordinal variables, we used weighted Kappa with quadratic weight.^14^ Discordant answers were weighted by squares of exact concordance deviations. To all statistics, we estimated a 95% confidence intervals.

We used the cut-off points suggested by Byrt et al., to classify level of stability of the answer: weak if 0 to 0.20, mild if 0.21 to 0.40, fair if 0.41 to 0.60, good if 0.61 to 0.80, very good if 0.81 to 0.92, and excellent if 0.93 to 1.00.^15^However, it is important to highlight that, in general, we recommended higher emphasis to numerical value because such scales can vary among authors.^16^

### Ethical and legal aspects of the study

Our study followed all ethical and legal requirements of the resolution 466/12 from the National Health Council/Brazilian Ministry of Health for research involving humans. This study was approved by INCA Ethics and Research Committee, protocol number 863,339 and CAAE: 33237314.2.0000.5274 - November 2014. The authors have no conflict of interests to disclose.

## RESULTS

Of 190 interviewed patients, 61.34% were men. Patients’ age ranged from 20 to 89 years, and mean age was 55 years (standard deviation - SD: 14.64); 61.86% of respondents declared to be white, 48.97% had complete or incomplete primary school, 57.73% were married or were in a consensual union, 74.21% were hospitalized for clinical treatment, and 73.70% were not diagnosed with distant metastasis at the time of the interview (Table 1).

**Table 1:** Clinical and demographic characteristics

Characteristics	n (%)
Sex	
Male	117 (61.34)
Female	73 (38.66)
Race	
White	118 (61.86)
Black/brown	72 (38.14)
Education	
Illiterate	3 (1.55)
Primary school	93 (48.97)
High school	67 (35.05)
College	27 (14.40)
Marital Status	
Single	41 (21.65)
Married	110 (57.73)
Widow/widower	39 (20.62)
Type of treatment	
Surgery	49 (25.79)
Clinical	141 (74.21)
Metastasis	
Yes	50 (26.29)
No	140 (73.70)
Age – Mean (standard deviation)	56.27 (13.61)

The five most common symptoms were dry mouth (60.31%), fatigue (58.5%), pain (56.19%), drowsiness (51.55%) and change in taste of food (48.97%). Five less frequent symptoms were problems with sexual desire or sexual activity (12.37%), problems to urinate (16.49%), itching (18.04%), difficult to swallow (18.56%), and diarrhea (21.13%) (Table 2).

**Table 2:** Summary of tests statistics of items in the Memorial Symptom Assessment Scale

Item	Prevalence	Frequency (%)				Intensity (%)				Discomfort (%)					Scores	
				
	n (%)	1	2	3	4	1	2	3	4	0	1	2	3	4	Mean	SD
Difficult to concentrate	48 (25.26)	6.28	58.64	27.22	4.19	45.83	37.50	14.58	2.08	76.29	2.06	14.90	3.61	3.09	1.73	0.79
Pain	109 (57.37)	9.17	29.36	36.70	23.85	11.93	42.20	33.03	12.84	44.85	0.00	27.90	10.31	17.01	2.47	0.87
Fatigue	113 (59.47)	11.50	35.40	37.17	15.93	35.40	45.13	12.39	7.08	42.27	2.58	36.60	12.37	6.19	1.91	0.87
Cough	88 (46.32)	19.32	42.05	29.55	10.23	55.68	34.09	10.23	0.00	55.15	8.76	29.40	3.09	3.61	1.55	0.68
Nervousness	87 (45.79)	11.49	47.05	27.59	14.94	31.03	39.08	20.69	9.20	54.12	2.06	32.50	6.19	5.15	2.08	0.94
Dry mouth	117 (61.58)	8.55	42.74	38.46	10.26	38.46	44.44	15.38	1.71	40.72	11.34	36.60	8.76	2.58	1.80	0.76
Sickness	76 (40.00)	21.05	43.42	23.68	13.16	40.79	36.84	21.05	1.32	60.82	0.52	26.20	8.25	4.12	1.83	0.81
Drowsiness	100 (52.63)	3.00	45.00	42.00	11.00	40.00	44.00	13.00	3.00	50.00	25.26	14.95	4.64	1.55	1.79	0.78
Numbness or tingling in hands/feet	80 (42.11)	10.00	37.50	21.25	31.25	50.00	37.50	12.50	0.00	59.28	7.22	26.80	4.12	2.58	1.63	0.70
Difficult to sleep	73 (38.42)	12.33	39.73	35.62	13.70	23.29	52.05	19.18	5.48	61.86	2.06	24.20	8.76	3.09	2.07	0.80
Feeling fullness	69 (36.32)	11.59	42.03	28.99	15.94	31.88	44.93	20.29	2.90	65.46	2.58	20.60	7.22	4.12	1.94	0.80
Problems to urinate	32 (16.84)	12.50	34.38	31.25	25.00	28.13	31.25	34.38	6.25	82.99	1.03	11.30	3.09	1.55	2.19	0.93
Vomiting	42 (22.11)	38.10	35.71	21.43	2.38	42.86	42.86	9.52	4.76	78.87	1.03	13.90	4.64	1.55	1.76	0.82
Shortness of breath	47 (24.74)	10.64	42.55	27.66	19.15	36.17	31.91	27.66	4.26	76.29	0.52	11.90	6.70	4.64	2.00	0.91
Diarrhea	41 (21.58)	21.95	39.02	34.15	4.88	36.59	39.02	19.51	4.88	78.87	1.55	15.50	2.58	1.55	1.93	0.88
Sadness	64 (33.68)	9.38	57.81	15.63	14.06	29.69	56.08	7.81	6.25	68.04	0.00	22.70	7.22	2.06	1.95	0.88
Sweat	65 (34.21)	13.85	53.85	30.77	4.62	27.69	44.62	24.62	3.08	65.98	10.82	15.40	5.67	2.06	2.03	0.81
Worries	88 (46.32)	14.77	44.32	20.45	22.73	26.14	55.55	10.23	7.95	53.61	2.58	29.90	11.34	2.58	2.07	0.94
Problems with sexual desire and sexual activity	24 (12.63)	8.33	33.33	33.33	25.00	16.67	41.67	16.67	25.00	87.63	0.00	8.80	2.58	1.03	2.50	1.06
Itching	35 (18.42)	17.14	51.43	31.43	2.86	40.00	28.57	31.43	0.00	81.96	1.03	13.90	0.52	2.58	1.91	0.85
Loss of appetite	83 (43.68)	9.64	31.33	27.71	30.12	20.48	42.07	28.92	8.43	57.73	5.67	23.70	7.73	5.15	2.29	0.93

SD: standard deviation.

In analysis or symptoms prevalence, most of items were within cluster symptoms more and less frequent found in PHYS H and PHYS L subscales. Differences were seen only in symptoms: cough was low prevalence in the subscale, and in our study, it was in the 8th position with 45.36% prevalence; and vomiting that belonged to subscale of symptoms with higher prevalence and, in our study, it appeared in the 26th position with only 21.56% prevalence.

We observed in degrees presented in three Likert-type scales that measured frequency, intensity, and discomfort for each item, the degree 2 was the most seen. The five items that most presented the degree 4 in the frequency scale were numbness or tingling (31.25%), loss of appetite (30.12%), problems to urinate (25.00%), problems with sexual desire and sexual activity (25.00%), and pain (23.85%), the five itens that most presented the degree 1 in the frequency scale were vomiting (38.10%), diarrhea (21.95%), nausea (21.05%), cough (19.32%) and itching (17.14%). The five items most presented degree 4 in intensity scale were problems with sexual desire and sexual activity (25.00%), difficult to swallow (18.92%), “I feel like I am not myself anymore” (20.78%), hair loss (18.92%) and irritation (13.64%). Items that most seen in the degree 1 were wounds in the mouth (57.78%), cough (55.68%), numbness or tingling in hands/feet (50.00%), difficult to concentrate (45.83%) and vomiting (42.86%). The five items most presented degree 4 in discomfort scale were pain (17.01%), constipation (6.70%), “I feel like I am not myself anymore” (5.67%), nervousness (5.15%) and loss of appetite (5.15%), those presenting more percentage in degree 0 were problems related to sexual desire and sexual activity (87.63%), problems to urinate (82.99%), itching (81.96%), difficult to swallow (81.44%) and hair loss (80.93%).

Therefore as an original instrument, MSAS-BR symptoms scale assess frequency, intensity and discomfort in 24 of 32 items and, in other 8 items, only intensity and discomfort were seen, because it was understood that symptoms such as hair loss, loss of appetite and wounds in the mouth were not considered relevant in terms of frequency because it was a continuous long-term situation.

Reliability of scale showed satisfactory results in tests-retest. Weighted Kappa values obtained for each item in the scale were high and varied from good to excellent, according to cut-off point adopted; the largest item was 0.96 and smaller was 0.69 (Table 3).

**Table 3:** Weighted Kappa statistics (test-retest) of answers to items that composed the scale and its subscales of Memorial Symptom Assessment Scale

Item	Mean* test		Mean* retest		Weighted Kappa^†^	
		
	Mean	SD	Mean	SD	k	95% CI
Difficult to concentrate	1.73	0.79	1.77	0.68	0.83	0.73-0.93
Pain	2.47	0.87	2.45	0.57	0.94	0.89-0.99
Fatigue	1.91	0.87	1.99	0.63	0.88	0.77-0.99
Cough	1.55	0.68	1.43	0.76	0.77	0.65-0.89
Nervousness	2.08	0.94	2.12	0.95	0.83	0.7-0.96
Dry mouth	1.80	0.76	1.68	0.89	0.79	0.68-0.9
Sickness	1.83	0.81	1.79	0.79	0.88	0.81-0.95
Drowsiness	1.79	0.78	1.67	0.78	0.84	0.74-0.94
Numbness or tingling in hands/feet	1.63	0.70	1.76	0.61	0.78	0.68-0.88
Difficult to sleep	2.07	0.80	2.15	0.64	0.86	0.75-0.97
Feeling fullness	1.94	0.80	1.87	0.76	0.93	0.88-0.98
Problems to urinate	2.19	0.93	2.08	0.89	0.81	0.7-0.92
Vomiting	1.76	0.82	1.89	1.03	0.73	0.62-0.84
Shortness of breath	2.00	0.91	1.92	0.63	0.79	0.67-0.91
Diarrhea	1.93	0.88	1.76	0.87	0.72	0.59-0.85
Sadness	1.95	0.88	1.94	0.54	0.97	0.95-0.99
Sweat	2.03	0.81	1.97	0.92	0.89	0.84-0.94
Worries	2.07	0.94	2.13	0.93	0.91	0.85-0.97
Problems with sexual desire or sexual activity	2.50	1.06	2.87	1.05	0.78	0.67-0.89
Itching	1.91	0.85	1.73	0.74	0.75	0.64-0.86
Loss of appetite	2.29	0.93	2.31	0.97	0.96	0.94-0.98
Dizziness	1.80	0.75	1.64	0.49	0.69	0.56-0.82
Difficult to swallow	2.25	1.20	2.24	1.19	0.94	0.92-0.96
Irritability	2.26	1.00	2.13	0.97	0.72	0.59-0.85
Wounds in the mouth	1.69	0.95	1.78	0.78	0.89	0.8-0.98
Change in taste of food	1.99	0.88	1.96	0.83	0.91	0.87-0.95
Loss of weight	1.84	0.86	1.76	0.97	0.83	0.71-0.95
Hair loss	2.27	1.12	2.38	1.03	0.85	0.72-0.98
Constipation	2.22	0.95	2.18	0.81	0.86	0.73-0.99
Swelling in arms and legs	1.97	0.88	1.75	0.85	0.73	0.62-0.84
“I feel like I am not myself anymore”	2.35	1.10	2.58	0.98	0.74	0.63-0.85
Changes on the skin	1.95	0.89	1.97	0.67	0.91	0.86-0.96

* Ranging from 1 to 4 points, ^†^ quadratic weight.

SD: standard deviation; 95% CI: 95% confidence interval.

We also obtained mean scores, standard deviations, weighted Kappa and 95% confidence intervals in test-retest for each subscale of PHYS H, PHYS L, PHYCH and GDI and mean among three domains, and all items. Weighted Kappa applied to these subscales also indicated good reliability related to cluster symptoms included. The subscale of PHYS domain had higher level of reliability (K=0.84) and domain of global distress index has lower level (K=0.78) (Table 4).

**Table 4:** Subscales statistics of inventory of symptoms in the Memorial Symptom Assessment Scale

Subscales	Domain	Items (n)	MSAS items	Test		Retest		Weighted Kappa	
		
				Mean score	SD	Mean score	SD	k	95% CI
PHYS H	Physics of high frequency	12	2, 3, 6, 7, 8, 11, 13, 21, 22, 26, 27 and 29	2.08	0.89	2.13	0.87	0.84	0.78-0.90
PHYS L	Physics of low frequency	14	4, 9, 12, 14, 15, 17, 19, 20, 23, 25, 28, 30, 31 and 32	2.84	0.38	2.77	0.46	0.81	0.75-0.87
PSYCH	Psychic	6	1, 5, 10, 16, 18 and 24	2.51	1.08	2.49	1.12	0.81	0.70-0.92
GDI	Glogal distress index	4/10	2, 3, 5, 6, 8, 16, 18, 21, 24 and 29	2.59	1.03	2.53	1.06	0.78	0.73-0.83
TMSAS	Global score	32	1 to 32	2.13	0.77	2.15	0.84	0.83	0.79-0.87

SD: standard deviation; GDI: Global Distress Index; MSAS: Memorial Symptom Assessment Scale; 95% CI: 95% confidence interval.

## DISCUSSION

The MSAS scale was developed as measurer for prevalence and characteristics of each group that include physical and psychological symptoms experienced by a number of oncology patients. This scale was validate in other countries such as China, Turkey, Sweden and Israel,^17-20^ and a high variability were seen in the most frequent symptoms. In analysis of symptoms prevalence observed in tests, similarities were seen in relation to high and less frequent symptoms seen in the validation study of the original instrument and its respective subscales PHYS H (most prevalent symptoms) and PHYS L (symptoms of relatively low prevalence). Different items, considered high, was cough, belonging to PHYS L with prevalence of 45.36%, and vomiting that belonged to PHYS H, considered relatively, and had prevalence of 21.65%. The most prevalence item was dry mouth (60.31%) and less prevalence was problems with sexual desire and sexual activity (12.37%). Other studies on MSAS scale test-retest reliability were not found in Brazil because of the recent semantic equivalence of the scale to Brazilian Portuguese, therefore, an comparisons of test-retest reliability and items prevalence are impossible in a national level.

In test-retest analysis (n=58), answers to items were stable, and concordance measured by weighted Kappa with quadratic weight ranged from good to excellent. We also observed similarities in values obtained in mean score between test-retests and weighted Kappa in analysis of subscales PHYS H, PHYS L, PSYCH, and GDI and total TMSAS. Such results show that MSAS-BR version was stable and presented significant results in weighted Kappa indexes, therefore showing high concordance between values and, therefore, adequate stability.

Zwart et al., revealed the time interval between tests that can be influence in test-retest reliability, in case of tests repletion in short time interval. Participants’ memory can influence results in second application and, therefore, falsely expand test-retest reliability, as well as changes in health status occurring during tests can also influence interviewees’ answers and reduce reliability of test-rest.^21^

## CONCLUSION

The present study, along with other investigations as to semantic validity, suggest a high stability of information collected by the instrument. In this sense, results achieved allow the use of instrument for the next stages of validation. Therefore, additional studies on validity will complete Memorial Symptom Assessment Scale psychometric assessment.
